# Investigating ongoing brain oscillations and their influence on conscious perception – network states and the window to consciousness

**DOI:** 10.3389/fpsyg.2014.01230

**Published:** 2014-10-30

**Authors:** Philipp Ruhnau, Anne Hauswald, Nathan Weisz

**Affiliations:** Center for Mind/Brain Sciences, University of TrentoTrento, Italy

**Keywords:** alpha oscillations, consciousness, near-threshold perception, network connectivity, Win2Con

## Abstract

In cognitive neuroscience, *prerequisites* of consciousness are of high interest. Within recent years it has become more commonly understood that ongoing brain activity, mainly measured with electrophysiology, can predict whether an upcoming stimulus is consciously perceived. One approach to investigate the relationship between ongoing brain activity and conscious perception is to conduct near-threshold (NT) experiments and focus on the pre-stimulus period. The current review will, in the first part, summarize main findings of pre-stimulus research from NT experiments, mainly focusing on the alpha band (8–14 Hz). It is probable that the most prominent finding is that local (mostly sensory) areas show enhanced excitatory states prior to detection of upcoming NT stimuli, as putatively reflected by decreased alpha band power. However, the view of a solely local excitability change seems to be too narrow. In a recent paper, using a somatosensory NT task, Weisz et al. (2014) replicated the common alpha finding and, furthermore, conceptually embedded this finding into a more global framework called “Windows to Consciousness” (Win2Con). In this review, we want to further elaborate on the crucial assumption of “open windows” to conscious perception, determined by pre-established pathways connecting sensory and higher order areas. Methodologically, connectivity and graph theoretical analyses are applied to source-imaging magnetoencephalographic data to uncover brain regions with strong network integration as well as their connection patterns. Sensory regions with stronger network integration will more likely distribute information when confronted with weak NT stimuli, favoring its subsequent conscious perception. First experimental evidence confirms our aforementioned “open window” hypothesis. We therefore emphasize that future research on *prerequisites* of consciousness needs to move on from investigating solely local excitability to a more global view of network connectivity.

## INTRODUCTION

Consciousness is one of the main enigmas of philosophy and has always been of great interest to experimental psychology. In recent years, understanding consciousness has also been a focus of cognitive neuroscience and can be described as one of the most important challenges in unraveling the neural basis of human cognition (Zeki, 2003; Dehaene et al., 2006; Lamme, 2006; Cohen and Dennett, 2011). Since advancing a mechanistic understanding of how phenomenological states emerge from neural activity patterns appears not currently feasible (hard problem, Chalmers, 1995), a popular strategy in cognitive neuroscience has been to first identify the so-called neural correlates of consciousness (NCC), defined as “the minimal set of neural events that give rise to a specific aspect of a conscious percept” (Crick and Koch, 1998).

Neural correlates of consciousness have been investigated with many different neuroimaging methods and electrophysiology. A strong focus initially lay on the differential response evoked by similar stimuli that were either reportable (i.e., consciously accessible) or not. To achieve a similar signal to noise ratio (i.e., equal amount perceived and non-perceived) these stimuli are oftentimes presented at the individual perception threshold. Therefore, a typically applied paradigm to investigate NCCs is the near-threshold (NT) paradigm. The design idea is simple; participants encounter a stimulus at a certain intensity such that it is equally likely to be perceived or not perceived, i.e., at perception threshold. The advantage of this design is that it is not the stimulus that changes from trial to trial but the quality of its perception (perceived; non-perceived). By investigating neural responses to stimuli that have been perceived and comparing them with those that have not been perceived, researchers have the unique opportunity to investigate neural processes based on (absent/present) consciousness.

A recent review has made a distinction between different NCCs (Aru et al., 2012). In order of appearance they are: Neural correlates of prerequisite processes (NCC-pr) – which are necessary for conscious access, yet they do not carry conscious content. Secondly, the NCC directly related to conscious experience – a neural correlate that, if present, would result in a specific conscious experience. Finally, consequences of conscious experience (NCC-co) represent after-effects of conscious experience (Aru et al., 2012). In typical event-related studies focusing on periods following the stimulus, a distinction between the “true” NCC and NCC-co is challenging due to strong overlaps; furthermore, even in the period following the stimulus onset there may be an additional impact of NCC-pr (Aru et al., 2012). In the current review we will focus on the prerequisite of consciousness (NCC-pr). We will emphasize the importance of ongoing oscillatory brain activity and suggest a network perspective when investigating prerequisites of consciousness. The importance of distributed network activity in supporting conscious perception has already been proposed by others (e.g., in the global workspace theory, Dehaene et al., 1998; Baars, 2005; and similarly, for a unitary percept within the micro-consciousness framework, Zeki and Bartels, 1999), however, the idea was confined to processing and propagation of already presented stimuli and has not been extended to the period preceding upcoming inflow.

The claim that pre-activated networks (or network connections) are required for conscious perception of NT stimuli has been made in a recent work by Weisz et al. (2014) and first evidence for this hypothesis has been provided. The “Windows to Consciousness” (Win2Con) framework proposes pre-established pathways, which enable the propagation of stimulus representations from sensory areas to higher order cognitive areas (such as prefrontal cortex) and thereby predisposing a conscious representation. We will describe the study by Weisz et al. (2014) in more detail in later parts of this review (see Windows to Conscious Perception) and we will also focus on the implications of the results for neuroscientific research of consciousness (see also other articles in this research topic).

In the first part of this review we will, however, summarize main findings related to the pre-stimulus period, that is, the time window preceding a stimulus. A main focus will be on the alpha band (8–14 Hz), which in terms of significance to, and attention of the neuroscience community has experienced a roller-coaster ride. Being the oldest rhythm described in the history of human electrophysiology, it has long been labeled as the brain’s “idling” response, since it is dominant during restful wakefulness and decreases during stimulation (Pfurtscheller and Silva, 1999). Current frameworks of alpha oscillations stress their functional significance as an inhibitory rhythm under top–down control (Klimesch et al., 2007). Alpha oscillations seem to serve as a mechanism to gate sensory information by inhibiting irrelevant or interfering regions and releasing inhibition from necessary areas (Jensen and Mazaheri, 2010). Furthermore, electrophysiological research found ongoing brain activity in the alpha range preceding upcoming stimuli that can predict whether the stimulus can be reported (i.e., consciously accessed) or not (e.g., Hanslmayr et al., 2007; van Dijk et al., 2008). These findings were remarkable and they have provoked quite a few following studies, summarized in the next paragraph.

## PRE-STIMULUS OSCILLATORY ACTIVITY PREDICTS CONSCIOUS PERCEPTION

There are a wide variety of studies investigating conscious access to low contrast stimuli. Since the late 1990s, many researchers have identified event-related components of conscious processing of similar stimuli (for a review see Dehaene et al., 2006). This approach, at least implicitly, holds that neural processes relevant to conscious processing are time-locked to the presentation of a stimulus. A few years ago, several studies challenged this notion finding that alpha band power preceding a low-contrast or NT stimulus was significantly lower when the stimulus would be reported as perceived as compared to when it was not perceived (Hanslmayr et al., 2007; Romei et al., 2008a; van Dijk et al., 2008). Importantly, alpha effects were largely constrained to posterior regions involved in visual processing, demonstrating that pre-stimulus effects can be regionally circumscribed and arguing against interpretations that overall arousal/wakefulness levels explain the differences. This general finding intrigued also, because it suggested that conscious perception can be determined by ongoing brain oscillations, which have conventionally been regarded as functionally irrelevant noise. The study and understanding of pre-stimulus oscillatory effects can now be considered as a central aspect in the investigation of NCC prerequisites.

A strong alpha rhythm in posterior regions predicts that an upcoming visual NT stimulus will not be perceived (Ergenoglu et al., 2004; Hanslmayr et al., 2007; Romei et al., 2008a; van Dijk et al., 2008; Busch et al., 2009; Wyart and Tallon-Baudry, 2009). Typically, these studies presented NT targets for a brief time (Busch et al., 2009; Busch and VanRullen, 2010; Lange et al., 2013) and masked them after offset to avoid sensory memory effects (van Dijk et al., 2008; Hanslmayr et al., 2009). Sustained power differences between subsequent perceived and non-perceived trials have been found in the second before the presentation of the NT stimulus. These pre-stimulus alpha decreases in perceived, as compared to non-perceived, trials showed a parietal to occipital topographical distribution, which was localized to visual cortex but also to parietal areas (Romei et al., 2008a,b; van Dijk et al., 2008; Lange et al., 2013). These localizations and the behavioral effects of alpha power increases (i.e., missing NT stimuli), as well as top–down modulated increases of alpha in task-irrelevant areas (Jensen et al., 2002; Thut et al., 2006; Jokisch and Jensen, 2007; Ciesielski et al., 2010), are supportive of the functional inhibition framework (Klimesch et al., 2007; Jensen and Mazaheri, 2010) wherein alpha oscillations are modulating the excitatory state of cortical regions. First invasive evidence for this hypothesis exists. A study on monkeys showed a correlation between alpha oscillations and neural firing, such that low alpha power goes together with an increase in neural firing (carrying the sensory representation, Haegens et al., 2011). Thus, alpha activity does directly affect excitability in local cortical areas. In the case of the visual NT design one might phrase as follows: a low contrast (NT) stimulus that enters the visual cortex during high alpha power does not (or only weakly) activate sensory neurons and – due to this failure – does not influence downstream areas essential for conscious perception.

Near-threshold experiments similar to the ones described above have also been done in the somatosensory modality. Alpha modulations in posterior regions and over somatosensory cortices have been found to modulate detection performance (Linkenkaer-Hansen et al., 2004; Sauseng et al., 2009; Schubert et al., 2009; Weisz et al., 2014). The relationship between alpha power (or in case of the somatosensory system, also called mu) and conscious perception, however, seems not to be as straightforward as in vision. Especially for primary somatosensory areas, alpha power and perception are more related in an inverted U-shape manner (lowest detection rates for low and high alpha power, highest detection rate with medium alpha power, Linkenkaer-Hansen et al., 2004). On the other hand, secondary somatosensory cortices as well as parietal areas show a very similar linear relationship as in the visual domain, increasing detection rates of NT stimuli with decreasing alpha power (Schubert et al., 2009; Weisz et al., 2014). Furthermore, evidence from research on a monkey showed a similar relationship between alpha in the somatosensory system and perception (Haegens et al., 2011). This study, however, applied a (difficult) tactile discrimination (perceptual decision-making) task. A recent study (Haegens et al., 2014) investigated thalamic and primary somatosensory cortex (S1) activity in an NT paradigm in two monkeys. This study showed a relation of alpha power in S1 to response bias, such that increased alpha power led to more overall reports of perceived stimuli. To conclude, even though not always completely consistent in terms of the pattern, alpha oscillations also seem to play a crucial role in the perception of tactile stimuli by modulating local excitability of somatosensory cortex.

Surprisingly, in the auditory domain a study well matched to the NT studies described above is still missing (for fMRI see Sadaghiani et al., 2009). This might be because measuring auditory alpha with non-invasive electroencephalography/magnetoencephalography (EEG/MEG) is challenging and therefore evidence on alpha in the auditory system is in general sparse (but see Frey et al., 2014; Strauß et al., 2014; Wilsch et al., 2014). However, several studies on auditory phantom percepts (Leske et al., 2013; Müller et al., 2013) – illusions without actual sensory stimulation, therefore a conceptual antagonist to NT stimulation – showed similar oscillatory modulations as in vision (for a recent comprehensive review on phantom percepts and oscillations see Lange et al., 2014). For instance, Leske et al. (2013) used the Zwicker tone, a brief auditory “afterimage” following the presentation of notch-filtered noise, to investigate the role of auditory alpha in illusory perception. Applying different types of experimental and analytic modulations they found that, generally speaking, lower oscillatory power in the alpha and beta range (10–20 Hz) coincided with a stronger perception of the illusion. Power modulations were most pronounced in Heschl’s gyrus, incorporating primary auditory cortex. Similar findings were provided for an illusion of music within presented noise (Müller et al., 2013), and an imbalance of cortical alpha is correlated with a pathological illusion: in tinnitus patients alpha power in auditory cortex regions is lower than in controls (Weisz et al., 2005; Hartmann et al., 2014). Thus, alpha oscillations play a crucial role in the generation of auditory illusions, resembling a similar inhibitory function as in other modalities.

It is worth noting that modulations of alpha power are not merely correlational. Studies using transcranial magnetic stimulation (TMS) showed a direct relationship between the (induced) alpha power and behavior (Romei et al., 2010). They presented lateralized visual NT stimuli and, using TMS, rhythmically stimulated the contra- or ipsilateral occipital cortex at 10 Hz. Participants detected substantially more stimuli when their ipsilateral occipital cortex was stimulated as compared to when the stimulation was contralateral. Furthermore, Romei et al. (2010) could show that the effect was specific to stimulation frequencies in the alpha range (10 Hz) but not to other frequencies (5 and 20 Hz).

As mentioned above, many studies on neural oscillatory activity showed an inhibitory function of the alpha band (Foxe and Snyder, 2011; Haegens et al., 2011; Mathewson et al., 2011; Spaak et al., 2012). This inhibitory function is not restricted to NT paradigms; in fact it has been shown that inhibition caused by alpha oscillation can even increase with stimulus intensity (Chaumon and Busch, 2014). Furthermore, alpha activity seems to be under top–down control (Jensen et al., 2002; Thut et al., 2006; Jokisch and Jensen, 2007; Ciesielski et al., 2010), such that task-irrelevant regions are inhibited by an increase of alpha power. It seems plausible that the neural system is inhibiting undesired routes, thereby actively opening specific gates to guide information through a distributed network architecture (Jensen and Mazaheri, 2010). To perform optimally, our neural system needs a strong inhibitor, which alpha activity seems to provide.

Oscillations are not only characterized by amplitude but also by phase. The idea (and in fact the scientific proof) that the phase of brain activity in relevant areas is directly correlated to how well that area can process information inflow is not new (Lindsey, 1952). In recent years, however, there has been an enhanced focus on this aspect. For instance, there is some evidence that visual perception samples the information inflow at a sampling rate of around 10 Hz (VanRullen and Macdonald, 2012). Thus, it is not surprising that, besides oscillatory power, the phase (or the phase distribution) before an actual stimulus can also be predictive of its perceptibility (Busch et al., 2009; Mathewson et al., 2009; Thorne et al., 2011). Effects of phase are not limited to vision but have also been shown in the somatosensory (Ai and Ro, 2014) and the auditory modality (Henry and Obleser, 2012; Neuling et al., 2012). Recent approaches using entrainment in the alpha range go so far as to ascribe phase the dominant role, causally related to behavior (Spaak et al., 2014). Crucially, however, phase effects are more closely aligned to the upcoming stimulus, and are typically found around stimulus onset (Mathewson et al., 2009; Thorne et al., 2011) or slightly before (Busch et al., 2009). While power modulations are more representative of a sustained excitability change in a task relevant region, phase seems to reflect increased excitability at certain points in time. Thus, it is not surprising that phase modulations are observed for expectation (Stefanics et al., 2010) and attention (Lakatos et al., 2008) when the timing of upcoming stimuli is known. A recent paper by Zoefel and Heil (2013) reminds us, however, that among all the recent phase-craze some caution may be warranted, by showing that contaminations by the evoked response can cause phase effects akin to those reported in the literature.

In summary, evidence from NT paradigms in different modalities showed a prerequisite NCC reflected in a modulation of cortical alpha power in task relevant, mostly local sensory areas. Low alpha power seems to be related to high excitability in sensory areas. Thus, in the case of reduced alpha power, an upcoming low contrast (NT) stimulus leads to an ignition of relevant neural assemblies in sensory areas and thereby a perceptual report is more likely. Local excitability, however, is not the only prerequisite for conscious perception as the Win2Con framework predicts (see Windows to Conscious Perception).

## ESSENTIAL NODES AND NETWORK PROPAGATION ARE NECESSARY FOR CONSCIOUSNESS

There are many findings showing specialized areas in the human brain representing the conscious percept and not just mere sensory stimulus input. For instance, the fusiform face area (FFA) and the parahippocampal place area (PPA) alternate in activity depending on whether the conscious percept is a face (FFA active) or a house (PPA active) with the sensory stimulation being identical (Tong et al., 1998). These areas, necessary for a specific content of consciousness, are sometimes called essential nodes (Zeki and Bartels, 1999). Many studies based on the Global Workspace theory (Baars, 1989) provided evidence that essential nodes are not responsible for a conscious percept by themselves, but that a propagation of the stimulus representation, mainly to a fronto-parietal network, is required for a stimulus to become conscious (Dehaene et al., 2001; Dehaene and Changeux, 2005; Nakamura et al., 2005).

For example, NT or masked visual stimuli that are not consciously accessible still elicit activations in respective lower level sensory brain regions (Dehaene et al., 2006). Yet, only stimuli with enough energy such that their representation is propagated up to higher-order areas lead to a re-activation of sensory areas (re-entrant/recurrent process) and “ignition” of the conscious process (Lamme and Roelfsema, 2000; Dehaene and Changeux, 2005; Dehaene et al., 2006). Furthermore, evidence from masking studies shows that the mask interrupts recurrent processes (Lamme et al., 2002). Several electrophysiological studies could demonstrate an enhanced functional connectivity for a consciously perceived stimulus as compared to the identical stimulus that went unnoticed (Palva et al., 2005; Melloni et al., 2007). Furthermore, multivariate decoding patterns applied to visual cortex can predict the presented stimulus, even in the absence of a conscious percept (Haynes et al., 2005). Thus, despite being an important component of the NCC, there is more to conscious perception than the activation of essential nodes. The model that initially proposed essential nodes, which are active for different aspects/features of the stimulus, is the model by Zeki and Bartels (1999) and Zeki (2001, 2003), which proposes micro-consciousness in each of the active nodes. All representations (nodes) exist independently. However, a unitary conscious percept can only exist when they are bound together (Bartels and Zeki, 2006). Thus, even though the basic representation of features is different, and no fronto-parietal areas are engaged *per se*, the crucial element that forms a unitary consciousness is again how individual parts of the percept are connected.

Most NCC research still follows the conventional approach in cognitive neuroscience, in studying local brain activity or functional connectivity states starting with the onset of the stimulus. This implicitly or explicitly degrades preceding and fluctuating ongoing brain activity as irrelevant noise. However, numerous works (see Pre-stimulus Oscillatory Activity Predicts Conscious Perception) assume and illustrate the high importance of ongoing brain activity for upcoming sensory stimulation. This suggests that presentation of sensory stimuli may fall into a period, described by local activation patterns and interregional functional connectivity patterns, which may be favorable or unfavorable for conscious perception (see also Windows to Conscious Perception). First evidence for the importance of network connections in ongoing activity comes from a recent study by Hipp et al. (2011). The study used an ambiguous stimulus, two bars that could either be perceived as bouncing off or passing each other, and investigated networks associated with the different percepts. They found that pre-stimulus activity in a fronto-parietal beta network was predictive of a bouncing or passing percept. This indicates that the content of consciousness is predisposed by ongoing activity in a large neuronal network.

In summary, whether consciousness is represented by activity in a global workspace or by the integration of information of multiple essential nodes with micro-consciousness, the common factor is the necessary interconnection, without which conscious access seems impossible. In the following chapter we will argue that this is similarly true for prerequisites of consciousness.

## WINDOWS TO CONSCIOUS PERCEPTION

The framework proposed by Weisz et al. (2014) added a new angle to the discussion of determinants of consciousness, or NCC-pr to stay within the terminology of Aru et al. (2012). The rationale of the framework is motivated by what is seemingly a paradox: if local excitability is not a sufficient explanation for conscious perception of a stimulus (e.g., Dehaene et al., 2006) in a post-stimulus period then why should local excitability be the crucial pre-stimulus ingredient of upcoming conscious perception? Indeed, as summarized convincingly by Dehaene et al. (2006), temporally early sensory activity, i.e., not caused by reentrant processes, does not differentiate between consciously perceived and non-perceived (identical) stimuli in post-stimulus intervals. This however would be expected if the main feature leading to subsequent perception of NT stimuli is the excitability level in sensory areas (e.g., neurons closer to firing threshold in V1 discharging following weak afferent input in the case of perceived stimuli).

**FIGURE 1:**
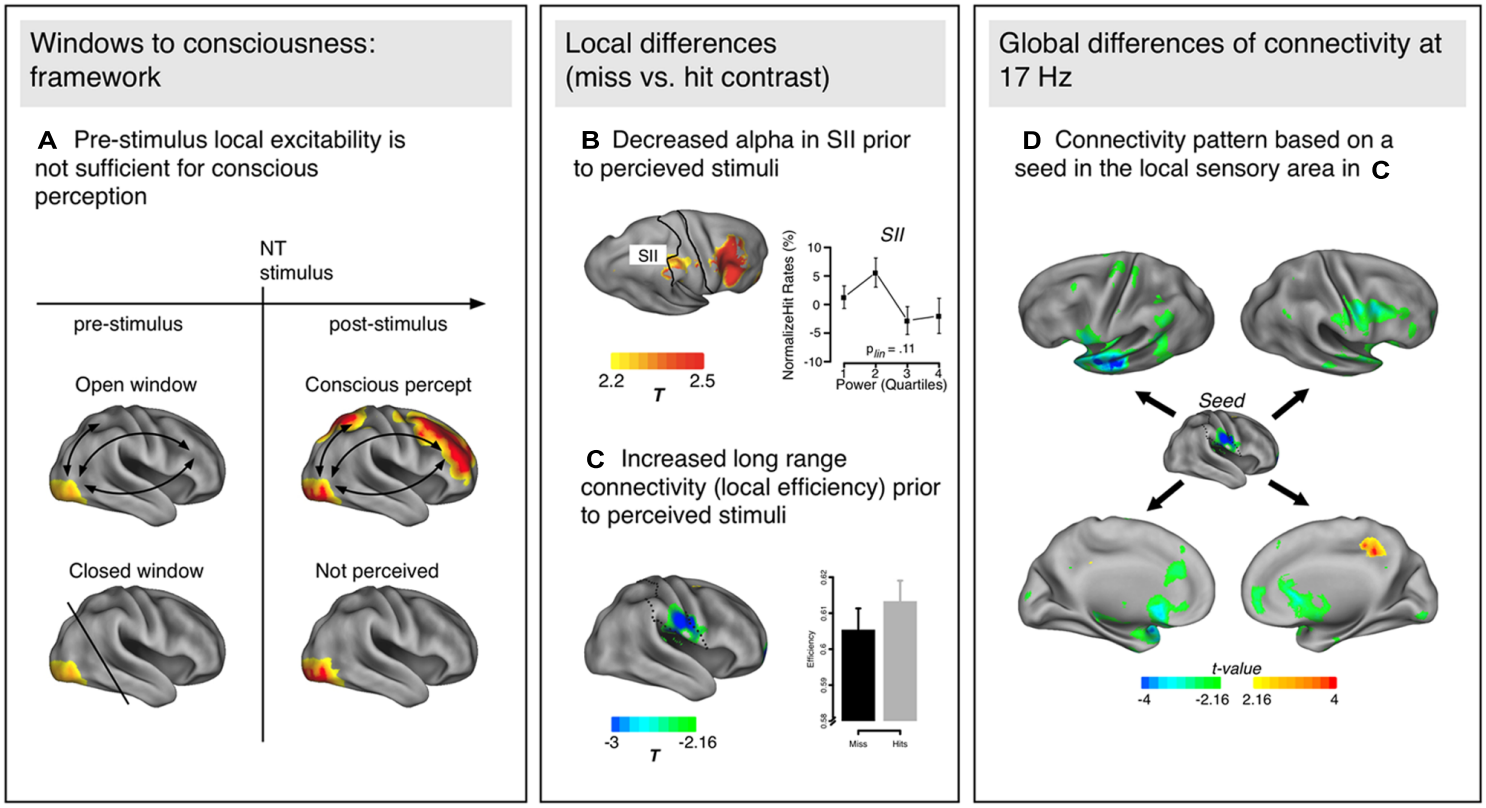
**(A)** Schematic representation of the Win2Con framework. Local excitability (yellow in visual areas) is not sufficient to predict the perception of a near-threshold (NT) stimulus, but additionally pre-activated network connections to higher order areas (e.g., prefrontal, parietal) are essential. **(B)** Local excitability changes in the somatosensory cortex (SII) as indexed by alpha decreases prior to perceived as compared to not perceived trials. **(C)** Additionally, local changes of connectivity (efficiency) have been observed in similar areas, such that efficiency was increased prior to perception of NT stimuli. **(D)** Topology of network modulations based on a seed in the area with the maximum connectivity modulation [SII in **(C)**]. The communication flow is increased with specific regions: mainly the left anterior temporal/inferior frontal areas, and other sensorimotor regions (e.g., premotor cortex, inferior parietal lobe). The power and connectivity results presented in **(B–D)** are from Figures 2–4 in Weisz et al. (2014).

Weisz et al. (2014) argue that in addition (or potentially even as a substitute) to local excitability, specific functional connections between sensory and higher order areas are predicting conscious perception (**Figure 1A**). It is therefore a matter of pre-established pathways forming windows to consciousness (thus the frameworks name: Win2Con) that allow a stimulus to be forwarded and consequently become conscious. Due to the abundance of pre-stimulus alpha effects in the literature, Win2Con builds upon the “gating-by-inhibition” hypothesis (Jensen and Mazaheri, 2010), which proposes that alpha activity can functionally inhibit (and release) nodes along the sensory pathway and thereby govern which content reaches consciousness and which is suppressed. It seems likely that alpha band activity plays a role in pre-established networks, although our framework makes no specific predictions about the frequency band involved. Crucially, the Win2Con framework predicts that the functional pathways activated in this gating process are already pre-activated, thereby constituting open windows to consciousness. There is some evidence that this is in fact the case.

In their study, Weisz et al. (2014) used an NT paradigm to investigate pre-stimulus activity in the somatosensory domain (see Wühle et al., 2010 for the original study investigating NCC). Two tactile stimuli were presented successively, one of them always supra-threshold the other one at perception threshold. Participants reported whether they perceived one or two stimuli. Contrasting pre-stimulus power for perceived and not perceived trials of the NT stimulus, the authors reported enhanced alpha in secondary somatosensory cortex [as well as middle frontal gyrus (MFG)] for subsequently not perceived NT stimuli (**Figure 1B**), thereby replicating the finding of local excitability changes in predicting the NT stimulus perception. More relevant within the proposed framework, they also analyzed network properties (global and local graph theoretical measures, see **Box 1**) between perceived and not perceived NT stimuli during the pre-stimulus period. On a global level, enhanced small-worldedness occurred around 17 Hz prior to not perceived NT stimuli compared to perceived ones. As small-worldedness is calculated as the ratio between clustering of a network and path length, both parameters can potentially influence the metric (more details on the relationship between different network properties can be found in **Box 1**). The authors investigated the nature of this effect by calculating global clustering and path length (as well as global efficiency) for the relevant frequency band and found that the enhanced small-worldedness prior to not perceived NT stimuli was due to a globally more clustered network compared to perceived ones. As global efficiency suggested shorter communication pathways prior to perceived stimuli, the authors concluded that networks preceding not perceived NT stimuli show more characteristics of local integration while those preceding perceived NT stimuli are more defined by global integration properties. These global differences underlying perceived and not perceived NT trials were further investigated using more fine-grained analyses on the frequency identified by the global analyses (17 Hz), namely local graph theoretical measures, which provide the location of specific network properties instead of a description applying to the entire network (**Figure 1C**). Confirming the global effects, increased local clustering and efficiency as well as reduced distance were observed for the secondary somatosensory cortex prior to perceived NT stimuli, presumably a key region for conscious somatosensory perception. Somatosensory cortex not only appears to be generally better integrated prior to perceived NT stimuli, but the communication flow is increased with specific regions (**Figure 1D**): mainly the left anterior temporal/inferior frontal areas and other sensorimotor regions (e.g., premotor cortex, inferior parietal lobe, etc.). Prior to not perceived NT stimuli, only the posterior cingulate gyrus showed enhanced connectivity.

**Box 1. Graph theory for electrophysiology: a very brief primer.**

Graph theory investigates different network properties of nodes (e.g., neurons, brain regions, or websites) and edges (e.g., axons, structural/functional connections, or hyperlinks). As the brain is a complex network of interconnected neurons, graph theoretical measures can also be applied to understand the dynamics of this complex system better (for reviews see Bullmore and Sporns, 2009; He and Evans, 2010; Sporns, 2012). Within the brain networks, the nature of nodes (i.e., voxels) and edges (i.e., connections) depend on different variables as measures and features of connectivity, brain mapping methods, and anatomical parcellation schemes (Rubinov and Sporns, 2010), all of which can influence the interpretation of the data.

With electrophysiology the following steps need to be taken to investigate frequency specific source space data with graph theory (see Bullmore and Sporns, 2009; and for details Weisz et al., 2014): First, after the projection of sensor space activity to source space (Schoffelen and Gross, 2009) a measure of association between individual nodes needs to be estimated (in Weisz et al., 2014, the imaginary part of coherence; for other approaches see Hipp et al., 2012), resulting in a node by node association matrix. Secondly, a binary adjacency matrix is generated by applying a threshold to the association matrix (for how to choose the threshold see van Wijk et al., 2010). The adjacency matrix (the graphical model of the brain network) can then be used to calculate network parameters of interest. Within the context of Win2Con the most interesting ones would be, for example:

• Node degree – The degree of a node reflects its overall connectedness to the network. It is the total number of connections linking this node to the rest of the network.
• Clustering – The clustering coefficient is based on the connections of the neighbors of a node. If the nearest neighbors are also connected to each other they form a cluster. Regular and complex networks, as opposed to random networks, typically show high clustering. It is important to note the difference of global and local clustering values. While global increases in clustering stand for segregation (forming of local, isolated “cliques”), local clustering is highly related to efficiency and therefore a measure of integration.
• Path length – Path length is the minimum number of edges that connect one node to another. Random and complex networks, as opposed to regular networks, typically show short path lengths.
• Efficiency – Efficiency is inversely related to path length, meaning that nodes with many short path lengths are overall more efficient. Together with path length, efficiency is an important measure of functional integration of a node. This measure is specifically interesting for the Win2Con framework as it can locally quantify the integration of, for instance, sensory areas in a larger network. Increased efficiency in sensory areas before perceived as compared to non-perceived trials is one of the key predictions of the framework.
• Small-worldedness – A network is small-worlded when its local clustering is high (i.e., a tendency to form local families exists) and path length connecting all nodes is short. It is calculated by dividing the clustering coefficient by the path length, both normalized by random network values of the same measure. This global measure can be a first indicator of network differences, which, however, need to be further investigated by evaluating clustering and path length (or efficiency) locally. Small-worldedness is specifically relevant for the Win2Con framework, as it quantifies the main predictions in one value: enhanced local activity (clustering) versus more efficient long-range connections (shorter path length). As mentioned in Windows to Conscious Perception, increased small-worldedness is, however, only a first indicator and local measures for clustering and efficiency have to be investigated to fully understand the nature of the effect.

The Brain Connectivity Toolbox (Rubinov and Sporns, 2010; https://sites.google.com/site/bctnet) offers a wide variety of functions in Matlab and C that calculate most of these measures.

The reported study by Weisz et al. (2014) provides first evidence for the Win2Con framework. Different sensory areas (here somatosensory regions) are differentially integrated within a network prior to the relevant stimulus, rendering its perception more or less likely. As proposed in the framework, local alpha modifications predicting the perception of an upcoming event (as suggested in the functional inhibition hypothesis) appear to depend on the level of integration between the region showing alpha modulations and the relevant network. In this sense, local excitability is necessary but not sufficient for conscious perception. Pre-established pathways within the relevant network are another prerequisite for NT stimuli to become consciously accessible. This is supported by findings of double dissociation of alpha modulations and graph theoretical measures: for example, while alpha power showed strong effects in the right MFG, effects of the graph theoretical measures were absent. *Vice versa*, significant changes of graph theoretical measures occurred in left anterior temporal/inferior regions without concomitant effects in alpha power. Accordingly, alpha power should not be mistakenly regarded as a proxy of functional connectivity.

Speaking of pre-established connections might trigger associations with other frequently investigated and reported networks such as, for example, the default mode network (Raichle et al., 2001). Findings from the study by Weisz et al. (2014) might nourish speculations that the default mode network constitutes such a network of pre-established pathways that modify the likeliness of subsequent conscious perception. In the current case of somatosensory NT stimulation, the posterior cingulate cortex, which supposedly is a core region of the default mode network, showed reduced connectedness with the somatosensory cortex prior to not perceived stimuli. Analogously, we want to point out that the idea of pre-established pathways relies on the assumption that, although globally increased connectivity might be a hint to an open window, it is not a general increase in connectedness of sensory regions that renders perception of an upcoming stimulus more likely, but connections with relevant areas, and integration of frontal and parietal regions that are crucially involved in the so-called global workspace (Dehaene et al., 1998).

## CONCLUSION AND FUTURE RESEARCH QUESTIONS

**FIGURE 2:**
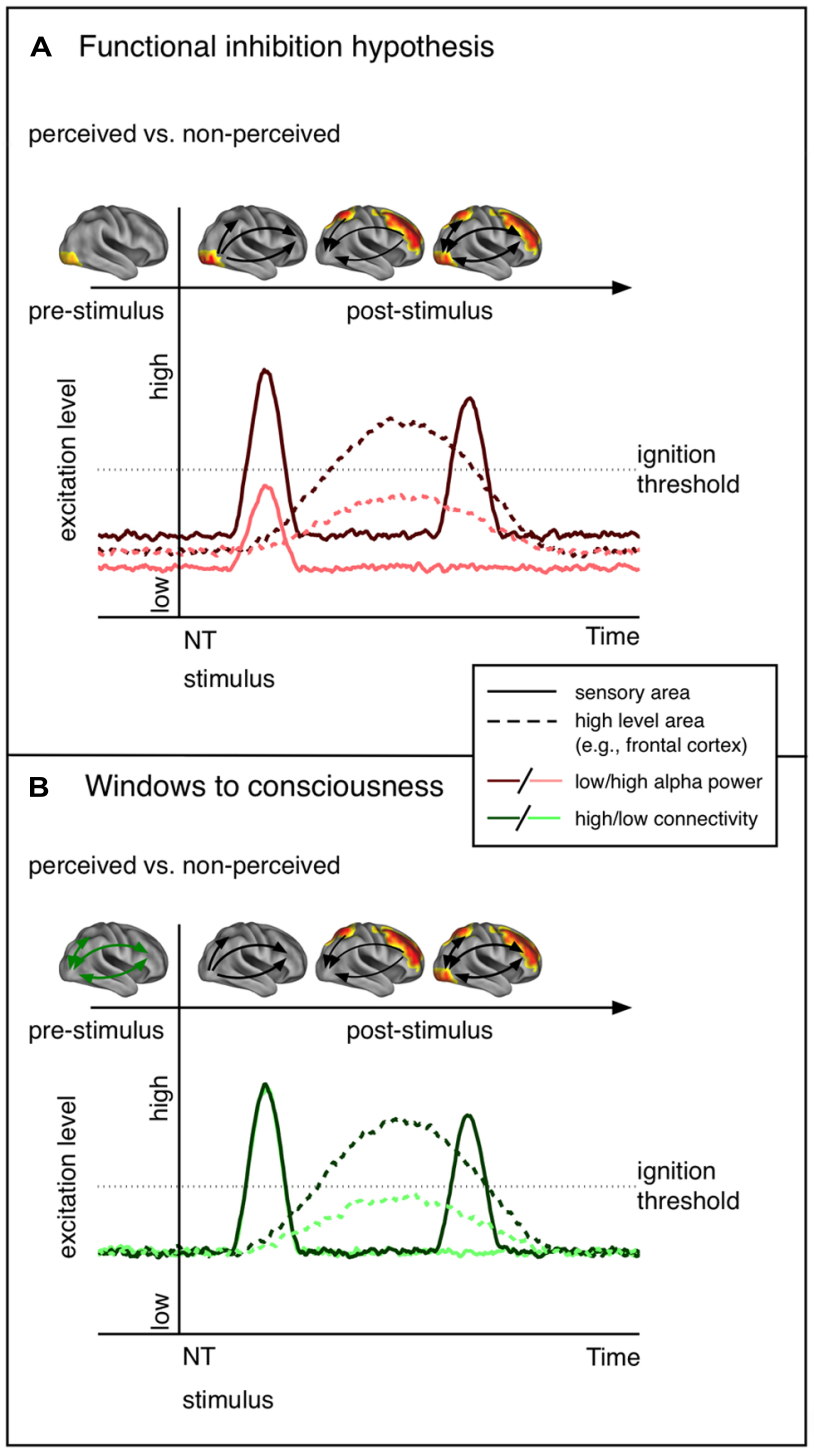
**Schematic representation of the predictions of the functional inhibition theory and the windows to consciousness framework.** The functional cortex images on top of each line plot show the hypothesized contrast of perceived vs. not perceived stimuli in terms of functional activity. **(A)** Functional inhibition hypothesis. Cortical excitability is modulated in sensory regions before NT stimulus presentation. Low alpha goes hand in hand with increased excitability and thus an NT stimulus entering sensory areas yields a stronger neural signal. The signal propagates to higher order areas, which send feed-back signals to sensory areas. The second peak in the sensory region reflects activity due to re-entrant activation. **(B)** Windows to consciousness framework. Under similar excitation levels, modulations of connectivity are expected. In case of pre-established pathways (high connectivity, dark green) early sensory activation, expected to be similar for perceived and not perceived trials, is propagated to high-level areas. This leads to re-entrant activation of sensory areas. Crucially, only late activity differences in sensory areas are expected.

In the current review we gave an overview of previous work regarding factors determining conscious perception with a focus on oscillatory alpha modulations prior to NT stimulation. In summary, lower alpha power in task relevant areas appears to be predictive of conscious perception of upcoming NT stimulation. However, it is worth mentioning that there is sparse evidence that the local excitability changes are bound to sensory regions as only a few studies have estimated the sources of alpha modulations (van Dijk et al., 2008; Lange et al., 2013; Weisz et al., 2014). Also, these local excitability alterations may not always be predictive of NT stimulus perception, at least in a linear manner (Linkenkaer-Hansen et al., 2004). Importantly, we claim that there exists an explanatory gap between the standard interpretation of pre-stimulus alpha effects that emphasize the role of local excitability, and the very sparse evidence showing an implication of respective regions at early latencies (see **Figure 2** for a schematic presentation of the predictions of the functional inhibition and the Win2Con framework). Consequently, we argued that local alpha power is indicative but not sufficient for conscious perception (at least for stimuli of weak intensity). As was proposed in a recent study (Weisz et al., 2014) introducing the Win2Con framework, a prerequisite for conscious perceptions is the integration of relevant areas in a broad network. Thus, only when local sensory excitability meets pre-established pathways, i.e., integration of the relevant area, will an NT stimulus be perceived.

There are still a few open questions that it is important to address: Firstly, as mentioned above, the actual location of the frequently reported alpha modulations prior to stimulus perception is often not known. Sources within sensory areas would have different theoretical implications than downstream sources: for example, the distinction between visual cortex and parietal regions is not always evident from sensor topographies alone. While the first would underline the importance of the current state of a specific sensory system, the latter may point toward a generalized increased attentive state. Therefore, supportive evidence should come from source localization efforts and/or intracranial recordings in animals or, for example, epileptic patients. Secondly, while building upon the alpha literature makes sense in light of the current evidence, the question remains whether power modulations in sensory areas are indeed necessary or whether the connectivity states alone are sufficient to predict conscious perception. Thirdly, and somewhat related, the exact relation between power and connectivity or local and inter-areal synchronization needs to be better understood. Fourthly, are pre-stimulus networks and post-stimulus networks comparable and, if yes, how do they relate? Fifthly, what kind of impact does pre-stimulus activity have on stimulus processing as indexed by evoked/induced brain activity? This can be investigated, for example, by using correlational approaches. Sixth, networks within the alpha–beta frequency range are not only theoretically good candidates but, as elaborated above, there is evidence for their involvement in conscious perception. Nevertheless, the question about other frequencies ranges and their interplay remains open. Finally, how causal are pre-stimulus connectivity patterns? This could be investigated using real-time experiments in which particular states (as previously identified in oﬄine studies) can be detected and stimuli presented at times of a specifically beneficial or detrimental state.

While the current review and introduction to our framework was geared toward the case of NT stimuli to illustrate our point, in real life many stimuli are clearly supra-threshold. In this case it is likely that sensory areas will be sufficiently activated to drive downstream areas, rendering the stimulus to be consciously perceived. However, it is also likely that trial-by-trial coupling fluctuations between sensory and downstream areas will influence post-stimulus activity patterns and thereby phenomenological experience. Apart from clever paradigms that allow for graded quantification of subjective experiences related to a stimulation, analysis methods would need to sufficiently develop to allow for the assessment of single-trial connectivity using MEG/EEG.

## Conflict of Interest Statement

The authors declare that the research was conducted in the absence of any commercial or financial relationships that could be construed as a potential conflict of interest.
